# Amitriptyline, clomipramine, and doxepin adsorption onto sodium polystyrene sulfonate

**DOI:** 10.1186/2008-2231-22-21

**Published:** 2014-01-22

**Authors:** Akram Jamshidzadeh, Fatemeh Vahedi, Omid Farshad, Hassan Seradj, Asma Najibi, Gholamreza Dehghanzadeh

**Affiliations:** 1 Pharmaceutical Sciences Research Center, Shiraz University of Medical Sciences, Shiraz, Iran; 2 Department of Pharmacology and Toxicology, Faculty of pharmacy, Shiraz University of Medical Sciences, Shiraz, Iran; 3 International Branch, Shiraz University of Medical Sciences, Shiraz, Iran; 4 Department of Pharmacognosy, Faculty of Pharmacy, Shiraz University of Medical Sciences, Shiraz, Iran; 5 Food and Drug Control Laboratory, Shiraz University of Medical Sciences, Shiraz, Iran

**Keywords:** Adsorption, Tricyclic antidepressants, Activated charcoal, Sodium polystyrene sulfonate

## Abstract

**Purpose of the study:**

Comparative in vitro studies were carried out to determine the adsorption characteristics of 3 drugs on activated charcoal (AC) and sodium polystyrene sulfonate (SPS). Activated charcoal (AC) has been long used as gastric decontamination agent for tricyclic antidepressants (TCA).

**Methods:**

Solutions containing drugs (amitriptyline, clomipramine, or doxepin) and variable amount of AC or SPS were incubated for 30 minutes.

**Results:**

At pH 1.2 the adsorbent: drug mass ratio varied from 2 : 1 to 40 : 1 for AC, and from 0.4 : 1 to 8 : 1 for SPS. UV–VIS spectrophotometer was used for the determination of free drug concentrations. The qmax of amitriptyline was 0.055 mg/mg AC and 0.574 mg/mg SPS, qmax of clomipramine was 0.053 mg/mg AC and 0.572 mg/mg SPS, and qmax of doxepin was 0.045 mg/mg AC and 0.556 mg/mg SPS. The results of adsorption experiments with SPS revealed higher values for the qmax parameters in comparison with AC.

**Conclusion:**

In vitro gastric decontamination experiments for antidepressant amitriptyline, clomipramine, and doxepin showed that SPS has higher qmax values than the corresponding experiments with AC. Therefore, we suggest SPS is a better gastric decontaminating agent for the management of acute TCA intoxication.

## Introduction

Tricyclic antidepressant (TCA) drug poisoning is a common cause of death from prescription drug overdose. Treatment includes aggressive supportive care, activated charcoal oral administration, alkalinization therapy, and management of arrhythmias, hypotension and seizures [1,2]. Activated charcoal (AC) has been used for gastric decontamination over the last century. It prevents absorption of substances in the gastrointestinal tract; thereby decreasing systemic absorption of potentially toxic agents [3]. Large reductions in drug absorption occur when AC is administered soon after drug ingestion [4]. AC is recommended for the treatment of TCA poisoning [5-7].

Sodium polystyrene sulfonate (SPS) is a potassium-binding resin used for the treatment of hyperkalemia [8]. SPS is not absorbed from the gastrointestinal tract. As the resin passes through the gastrointestinal tract, the resin removes the potassium ions by exchanging it for sodium ions. In clinical practice, SPS is often mixed with cathartics such as sorbitol to prevent constipation, which is sometimes seen with SPS [9-11].

The purpose of this in vitro investigation is: (a) to determine whether SPS is effective for the adsorption of amitriptyline, clomipramine, and doxepin; (b) to show whether SPS is better than AC in decreasing the free drug concentrations of TCAs; and (c) to determine the time needed for the complete adsorption process.

## Materials and methods

### Apparatus

A Cecil, UV–VIS spectrophotometer (England) with 1 cm quartz cells was used for all absorbance measurements.

### Chemicals and reagents

Amitriptyline and doxepin were obtained from Darou Pakhsh Pharmaceutical Company (Iran) and clomipramine was obtained from Shahre Darou Pharmaceutical Company (Iran). AC (Asche ~ 5%; Fe < 0.3%; Particle size 75% < 40 μ; Loss on drying ~ 10%) and SPS were obtained from Modava Pharmaceutical Company (Iran). All other chemicals were of analytical reagent grade purchased from Merck (Germany) unless otherwise specified. Distilled water was used to prepare all solutions. Acidic medium pH 1.2, simulating gastric environment was prepared by adding 12 M hydrochloric acid to water. The final pH was checked by pH meter and kept at 1.2 during the experiments.

### Standards for calibration curve

Standard stock solutions of amitriptyline, clomipramine, and doxepin were prepared in distilled water at the concentration of 0.25 mg/ml. Working standard solutions were prepared by suitable dilution of standard stock solution with distilled water.

### Solutions for adsorption experiments

The sixteen working standard stock solutions of 0.25 mg/ml of drugs (amitriptyline, clomipramine, and doxepin) were prepared in the acidic medium pH 1.2 in 100 ml volumetric flasks, then 50–1000 mg AC was added to 100 ml of each working standard solution. Similarly, The fifteen working standard stock solution of 0.25 mg/ml of drugs (amitriptyline, clomipramine, and doxepin) were prepared in the acidic medium pH 1.2 in 100 ml volumetric flasks, and 10–200 mg SPS was added to 100 ml of each working standard solution. SPS was first washed carefully with distilled water to remove interference materials and then dried in oven at 80°C for 24 hours.

The suspensions were shaken at 37°C for 30 minutes to establish equilibrium. Each sample was filtered through a paper filter. 1 ml of the resulting filtrate was transferred to a tube and diluted with 9 ml hydrochloric acid (pH 1.2). The values of absorbance were measured at 239 nm for amitriptyline, 252 nm for clomipramine and 292 nm for doxepin. This process was repeated 3 times for each sample.

In order to study the kinetic profile of the adsorption process of each drug (amitriptyline, clomipramine and doxepin) onto AC and SPS, various incubation times were tried. 25 mg of each drug (amitriptyline, clomipramine and doxepin) were separately transferred to a 100 ml volumetric flask and 500 mg AC or 50 mg SPS were added. The suspensions were shaken at 37°C with different incubation times (20, 40, 60, 80, 100 and 120 min).

### Estimation of drug adsorption parameters (data analysis)

Several isotherm equations have been used for the equilibrium modeling of adsorption systems. Among these, the most widely used are the Langmuir and Freundlich isotherm equations. In our study, the Langmuir adsorption isotherm was used to estimate adsorption parameters: qmax was defined as the maximum quantity (mg) of drug adsorbed per mg AC or SPS. The affinity constant (K) in L/mg quantifies the interaction between drug and AC or SPS. The adsorption parameters were calculated by linear least-squares fitting of the following expression of the Langmuir model to the experimental data (Equation 1): [12].

$$\frac{1}{\mathrm{qe}}=\frac{1}{\mathrm{qmax}}+\frac{1}{\mathrm{Kqmax}}\;\frac{1}{\mathrm{Ce}}$$

qe = amount of drug adsorbed by AC or SPS (mg/mg) Ce = drug concentration in the solution at equilibrium (mg/L)

## Results

### Drug adsorption onto AC and SPS

The results of adsorption experiments with SPS revealed higher values for the qmax parameters in comparison with AC. This defines a strong binding of amitriptyline, clomipramine, and doxepin onto SPS in comparison with AC (Table 1).

**Table 1:** **Adsorption parameters (qmax, affinity constant K and R**^**2**^**) of amitriptyline, clomipramine and doxepin onto activated charcoal (AC) and sodium polystyrene sulfonate (SPS)**

	**Langmuir isotherm method**			
		**qmax**	**K**	**R**^**2**^
	Amitriptyline	0.055	3.469	0.972
AC	Clomipramine	0.053	3.464	0.945
	Doxepin	0.045	0.777	0.956
	Amitriptyline	0.574	0.702	0.904
SPS	Clomipramine	0.572	0.492	0.975
	Doxepin	0.556	0.225	0.914

**Figure 1:**
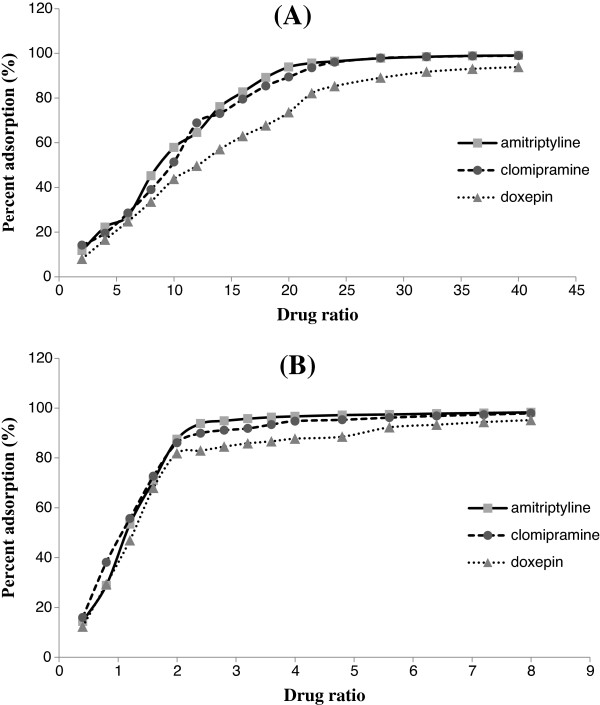
Amount of drug adsorbed onto A: activated charcoal (AC) and B: sodium polystyrene sulfonate (SPS).

Figure 1A-B presents the amount of drug adsorbed onto AC and SPS. For AC, a minimum of 90% drug adsorption onto the adsorbent was reached when the adsorbent: drug ratios were 18: 1, 20: 1 and 28: 1 for amitriptyline, clomipramine, and doxepin, respectively. For SPS, a minimum of 90% drug adsorption to the adsorbent was reached when the adsorbent: drug ratios were 2: 1, 2.4: 1, and 4.8: 1 for amitriptyline, clomipramine, and doxepin, respectively.

**Figure 2:**
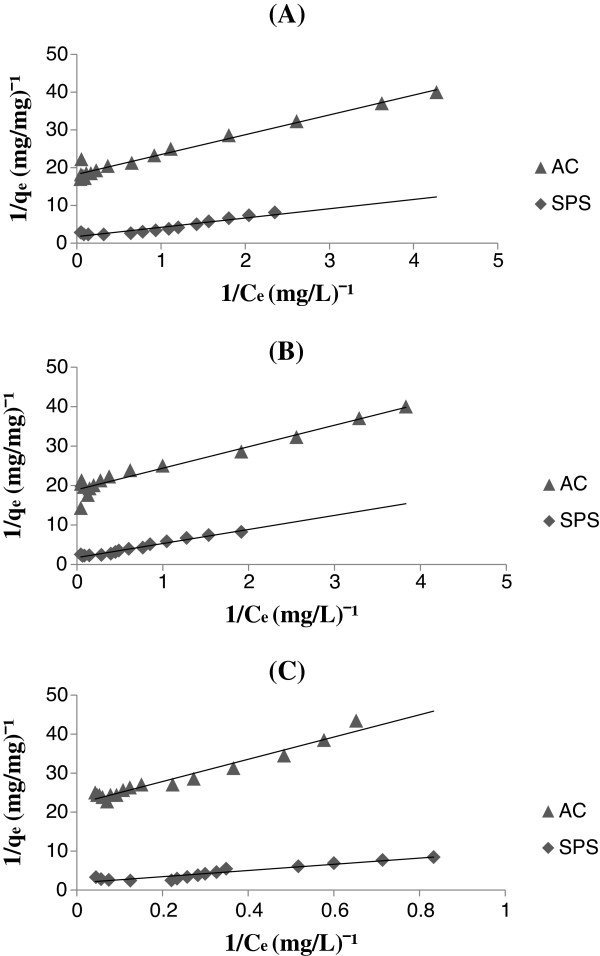
Langmuir plots of the adsorption of amitriptyline (A), clomipramine (B) and doxepin (C) onto activated charcoal (AC) and sodium polystyrene sulfonate (SPS).

Figure 2 shows Langmuir plots of the adsorption of amitriptyline (A), clomipramine (B) and doxepin (C) onto AC and SPS. These plots were used to determine the K and qmax values.

### Effect of contact time

**Figure 3:**
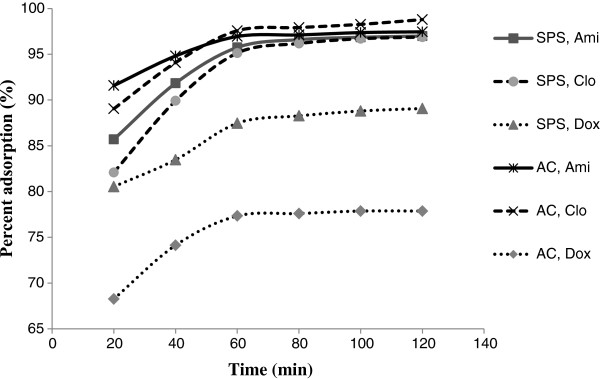
Drug mass ratio of activated charcoal (AC) and sodium polystyrene sulfonate (SPS).

As shown in Figure 3, for an adsorption experiment with an AC: drug mass ratio of 20:1 and SPS: drug mass ratio of 2:1, the maximum adsorptions were established after 60 minutes. The plots indicate that the rate of drug adsorption increases rapidly in the beginning, but become very slow at the end.

## Discussion

The Initial treatment of an acute overdose includes gastric decontamination of the patient [13]. AC has the capacity to adsorb a wide range of substances and organisms [14]. AC can bind with many drugs such as amitriptyline, clomipramine, and doxepin, and it is not absorbed in the gut. In our study, SPS, a cation-exchange resin, showed an effective adsorption of amitriptyline, clomipramine, and doxepin presumably based on their cationic properties.

Previous reports indicated that molecular structures of drugs are important factors in the adsorption mechanism. Higher adsorption is usually seen in aromatic compounds compared to aliphatic compounds of similar molecular size, and in branched-chain molecules compared to straight-chain molecules. Tricyclic antidepressants, having an aromatic containing tricyclic structure and a branched-chain structure, have been described to be well adsorbed to AC in vitro [15]. Most drugs are weak acids or bases. Weak acids and bases are weak electrolytes meaning that they are only ionized to a small extent (less than 1%) in neutral solution. The final pH of the drug solution will depend upon the pK_a_. At pH 1.2 and 7.2, the amine group of amitriptyline (pK_a_ 9.4), clomipramine (pK_a_ 9.4), and doxepin (pK_a_ 9.0) becomes protonated and these ionized substances adsorb poorly [16,17].

In its ionized form, adjacent molecules will repel each other when adsorbed on the AC surface because they carry the same electrical charge. The result is a lower density of drug packed together on the surface; therefore, the maximum adsorption capacity will be reduced compared to a non-ionized drug [15].

Ion exchange resins (IER) are insoluble polymers that contain acidic or basic functional groups and have the ability to exchange counter-ions within aqueous solutions surrounding them. Based on the nature of the exchangeable ion of the resin as a cation or anion form, it is classified as cationic or anionic exchange resins, respectively. Cation exchange resins contain covalently bound negatively charged functional groups and exchanges positively charged ions. SPS is a strongly acidic ion-exchange resin and is used to treat hyperkalemia [18]. SPS has been promising in animal and healthy human volunteers to reduce Li absorption and promote its elimination [19]. Previous studies showed that calcium polystyrene sulfonate, a cation-exchange resin, could adsorb imipramine, clomipramine, mianserin, trazodone, and ciprofloxacin based on their cationic properties [20].

The chemical behavior of this resin is similar to that of a strong acid. This resin is highly ionized in both the acid (R-SO_3_H) and salt (RSO_3_Na) forms of the sulfonic acid group (−SO_3_H). The hydrogen and sodium forms of strong acid resins are highly dissociated, and the exchangeable Na^+^ and H^+^ are readily available for exchange over the entire pH range. Consequently, the exchange capacity of strong acid resins is independent of the solution pH [18].

In our study, SPS showed to effectively adsorb amitriptyline, clomipramine, and doxepin. This fact can be attributed to the adsorption of protonated amine groups of these drugs onto SPS surface after exchanging with sodium.

The initial faster rate of drug adsorption may be explained by the large number of sorption sites available for adsorption. For the initial bare surface, the sticking probability is large, and consequently adsorption proceeded with a high rate. The slower adsorption rate at the end is probably due to the saturation of active sites and attainment of equilibrium (Figure 3).

## Conclusion

These adsorbents showed the adsorption characteristics by physical adsorption (AC) and complex formation of ion-exchange resin (SPS). In vitro experiments for antidepressant amitriptyline, clomipramine, and doxepin indicated that sodium polystyrene sulfonate (SPS) has higher qmax values than activated charcoal (AC). Therefore, we suggest SPS is a better gastric decontaminating agent for the management of acute TCA intoxication.

## Competing interests

The authors declare that they have no competing interests.

## Authors’ contributions

AJ, FV, OF, HS, AN and GD carried out the amitriptyline, clomipramine, and doxepin adsorption onto sodium polystyrene sulfonate and drafted the manuscript. All authors read and approved the final manuscript.
